# Urinary Diversion with Left Sided Ileal Conduit for Neurogenic Bladder in a Patient with Sacral Agenesis and Situs Inversus: A Case Report

**DOI:** 10.31729/jnma.v63i292.9259

**Published:** 2025-12-31

**Authors:** Sushna Khanal, Nirav Ojha, Aashis Poudel, Aashish Giri, Himal Karki, Chandra Shekhar Yadav, Kabir Tiwari

**Affiliations:** 1 Department of General Surgery and Urology, Kirtipur Hospital, Kathmandu.; 2 Nepal Red Cross Society, Kathmandu; 3 Patan Academy of Health Sciences, Lalitpur

**Keywords:** *neurogenic bladder*, *situs inversus*, *urinary diversion*

## Abstract

Sacral agenesis is a rare cause of neurogenic bladder which leads to urinary incontinence, recurrent urinary tract infections, and progressive renal dysfunction. The coexistence with situs inversus adds anatomical complexity, posing challenges in clinical and surgical management. We reported a case of a 23-year-old male with sacral agenesis and situs inversus presenting with incontinence and recurrent urinary tract infections. The patient was non-compliant to clean intermittent selfcatheterization. He underwent successful urinary diversion with a left-sided ileal conduit. Postoperative recovery was uneventful, with improved renal parameters and quality of life. Urinary diversion using a left-sided ileal conduit is a rare but feasible option for treatment when right-sided diversion is contraindicated. This case highlights that left-sided ileal conduit is a safe and effective option in managing neurogenic bladder in the context of sacral agenesis and situs inversus, underscoring the need for early recognition and individualized surgical planning in congenital anomalies.

## INTRODUCTION

Sacral agenesis is a congenital cause of neurogenic bladder, characterized by partial or complete absence of the lower sacral vertebral bodies, with prevalence 2.6 per 100,000 live birth.^1^ Neurogenic bladder presents with incomplete emptying, urgency, frequency, incontinence, and recurrent urinary tract infections and may cause renal failure. Management includes behavioural techniques, clean intermittent catheterization, urinary diversion.^2^ Sacral agenesis is occasionally associated with situs inversus, complicating management.^3^

We report a case of a 23-year-old male with neurogenic bladder due to sacral agenesis with situs inversus treated with urinary diversion with left sided ileal conduit, reported as per SCARE guidelines 2025.^4^

## CASE REPORT

A 23-year-old male presented to the outpatient department of our centre with complaints of burning micturition for 1 week and stank pain for 3 days. He had no history of fever, vomiting or hematuria. He had urinary leakage since childhood. The caretaker had to change his partially to fully soaked diaper 2-3 hourly. Upon further inquiry, at his age of four his parents brought him to a children’s hospital for his urinary incontinence. He also had a history of right ureteric reimplantation for grade IV vesicoureteric reflux in his childhood.

At 14 years of age, his parents took him to a hospital for the same complaint. He was evaluated with Retrograde Urogram (RGU) and Micturating Cystourethrogram (MCUG); and found to have absent sacrum beyond S2, small capacity of urinary bladder with pine-cone appearance, reflux in the left ureter. Ultrasonography (USG) of abdomen and pelvis showed features suggestive of situs inversus and normal bilateral kidney and no hydronephrosis. The impression of partial sacral agenesis with neurogenic urinary bladder and left grade I Ultrasonography (USG) of abdomen and pelvis vesicoureteral reflux (VUR) with situs inversus was made. He was advised for for clean intermittent catheterization (CIC) or long term suprapubic catheter.

Throughout his adulthood, he adapted with adult diapers, leading to late presentation. He couldn’t receive treatment due to financial burden, lack of awareness and family responsibilities.

He was born of a non-consanguineous marriage, by normal vaginal delivery at term following an uneventful pregnancy of a non-diabetic mother.

On general physical examination, he was well built. On abdominal examination, a linear surgical scar of the previous surgery was noted over the left supra pubic region with normal external genitalia. Neurological and musculoskeletal examinations were normal.

**Figure 1:**
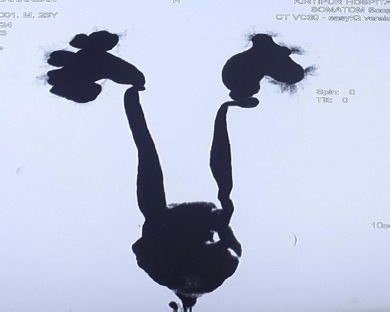
Bilateral moderate hydroureteronephrosis on Computed Tomography Urogram.

**Figure 2:**
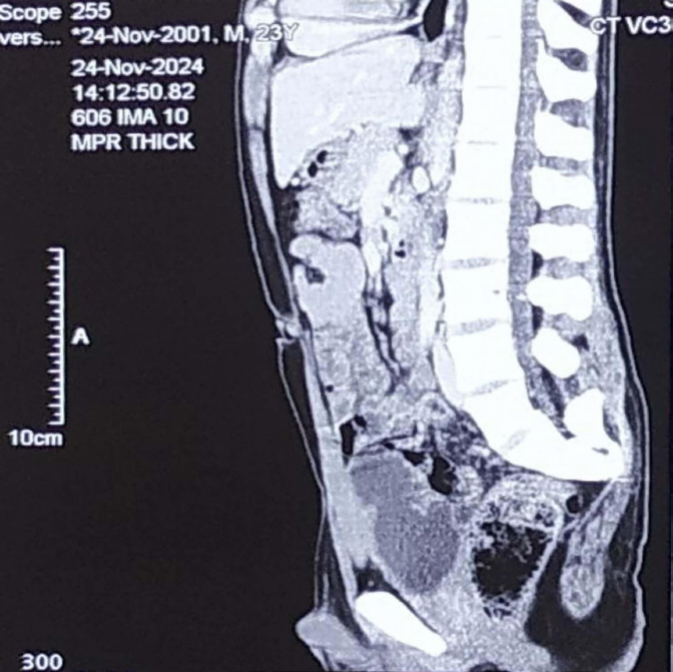
Computed tomography showing sacral agenesis.

On further investigation laboratory investigation revealed Blood urea: 8 mmol/L (2.5-7.5), Serum Creatinine: 130νmol/L (40110), Sodium: 138 mEq/L, Potassium: 3.9 mEq/L and urine routine revealed plenty of pus with no growth in urine C/S. On radiological investigation, USG abdomen and pelvis showed bilateral moderate hydroureteronephrosis with thickened trabeculated urinary bladder wall. Computed Tomography Urogram also showed bilateral moderate hydroureteronephrosis (Figure 1), thickened irregular urinary bladder wall and multiple diverticuli and normally excreting bilateral kidney (Figure 2). He was admitted with a diagnosis of neurogenic bladder with renal impairment.

After a week he was symptomatically better and was advised for CIC but he did not comply. Hence, he was discharged with silicone foley catheter in situ and asked to follow up. He was on regular follow up. Despite foley catheter in situ for 3 months there was increasing hydronephrosis and so surgery was planned.

**Figure 3:**
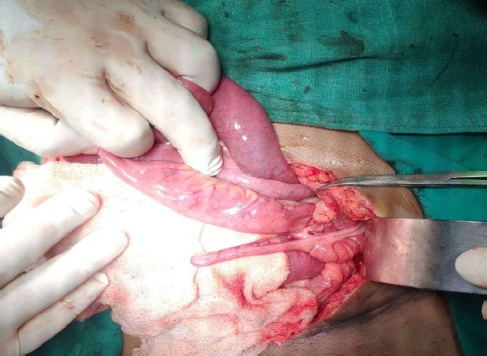
Intraoperative picture showing ileal segment isolated on the left side 15cm proximal to ileocaecal junction and appendix (blue arrow)

**Figure 4:**
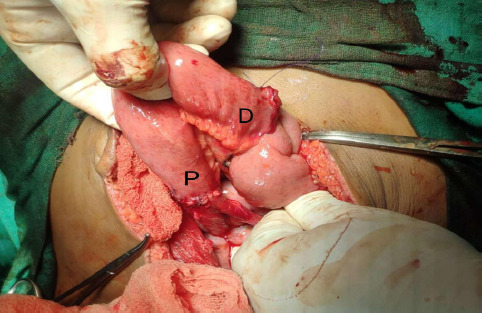
Intraoperative picture showing Proximal(P) and distal(D) side of ileal conduit.

The patient was admitted, pre-operative investigations were done and adequate bowel preparation was done. General anesthesia was given. On cystoscopy, small capacity bladder, sacculations and diverticulation suggestive of chronic obstructive changes were noted. Under all septic conditions, the abdomen was opened using low midline incision. Bilateral ureter was isolated and separated from its junction with the urinary bladder. About 15cm of terminal ileal segment was isolated on its mesentery, about 15 cm away from ileo-caecal junction which was on the left side of the abdomen (Figure 3). Bilateral ureters were approximated and the medial border of both ureters anastomosed using Wallace-I method thus, creating a ureteral plate. Double J (DJ) stent was kept bilaterally. The ureteral plate was anastomosed with the proximal end of the ileal conduit (Figure 4). Stoma was fashioned on the left side of the abdomen and a 16F Foley’s catheter was placed in the conduit.

The intraoperative period was uneventful with less than 500ml of blood loss not necessitating a blood transfusion. Perioperative period was smooth as well, he passed status on the second postoperative day and stool was passed on the fifth postoperative day. His renal function test remained normal throughout this period. His hospital stay was 9 days, bilateral DJ stents were removed on 14 th postoperative day.

**Figure 5:**
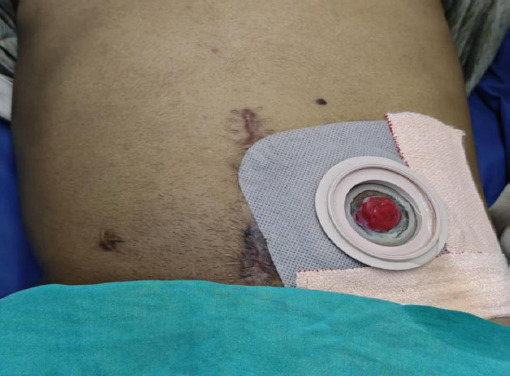
Postoperative wound with left sided ileal stoma(blue arrow)

On follow-up after 5 months, his quality of life has drastically improved and the stoma care is being done with no stoma leakage or superficial skin changes (Figure 5). His His USG abdomen and pelvis revealed bilateral mild hydronephrosis. He has been changing his stoma without any complications and the patient has returned to daily activities, reporting improvement in quality of life.

## DISCUSSION

We report a case of a 23-year-old male with sacral agenesis and situs inversus, who presented with neurogenic bladder and recurrent UTIs. He underwent successful urinary diversion via a left-sided ileal conduit.

Sacral agenesis results from failure in the development of the caudal mesoderm during early embryogenesis, within the first 4 weeks of gelation.^5^ It represents a spectrum of caudal regression syndrome (CRS), a term coined by Duhamel in 1961 to describe the developmental failure of the lower spine and spinal cord.^6^ CRS and sacral agenesis are often used interchangeably. This condition is associated with maternal diabetes, periconceptional smoking, and other congenital anomalies such as anorectal malformations, limb abnormalities, and genitourinary defects.^7^ However, our patient had no known teratogenic exposures or familial predisposition.

Neurogenic bladder in CRS results from impaired neural innervation of the bladder and sphincter, leading to dysfunction in storage and voiding phases. It can occur due to lesions at various levels of the central and peripheral nervous system and is commonly seen in spina bifida, spinal lipomas, myelomeningocele, and caudal regression.^8^

Patients with sacral agenesis commonly present with incontinence, urgency, frequency, nocturia, and recurrent UTIs and have a higher risk of VUR.^8^ The condition is frequently underdiagnosed, often due to limited healthcare access, socioeconomic constraints, and poor awareness, leading to delayed presentation.^9^ The patients may present in adulthood with hydronephrosis, renal failure, UTIs, urolithiasis, bladder cancer, and sexual dysfunction.^6^

Initial management of neurogenic bladder starts with conservative measures like clean intermittent selfcatheterization, anticholinergics, and bladder training, which help lower intravesical pressure and protect renal function.^6^ However, these often fail in cases with poor compliance, bladder abnormalities, or extensive sacral agenesis, thereby requiring surgical intervention. Our patient was non-compliant to conservative management which was due to longstanding psychosocial adjustment and socioeconomic barriers, a common issue in low-resource settings.^9^

Therefore, urinary diversion became the most viable option. Urinary diversion refers to the surgical rerouting of urine flow from its normal pathway. The first urinary diversion was attempted in 1852, and the ileal conduit technique—one of the most commonly employed forms—was introduced by Zaayern in 1911.^6^ Among the various types of urinary diversion, the ileal conduit is an incontinent, non-reservoir diversion. In our patient, due to situs inversus, surgical anatomy was mirrored. This method is technically simpler and associated with fewer complications compared to continent diversions, making it ideal in resource-limited settings or in patients who are not suitable candidates for self-catheterization.^6^

The literature on left-sided ileal conduit urinary diversion is limited. Nakamura et.al. reported successful construction of a left-sided ileal conduit for urinary diversion in a 75-year-old woman undergoing radical cystectomy, due to inability to use the standard right lower abdominal site due to scar hernia from previous surgery.^10^ Qakici et al. reported successful construction of a modified left-sided ileal conduit for urinary diversion in a 68-year-old man following urgent radical cystectomy, because of a prominent short left ureter, with the conduit passed under the sigmoid mesentery to accommodate ureteral length.^11^ Rodrigues et al. reported successful left-sided ileal conduit construction in an 82-year-old man with situs inversus totalis and muscle-invasive bladder cancer, where the stoma was placed on the left due to mirror-image anatomy, marking the first documented case of urinary diversion in this rare congenital condition.^12^

Our patient’s postoperative course was uneventful, and follow-up showed normal renal function, absence of UTIs, and cessation of adult diaper use, indicating significant quality-of-life improvement. Similar outcomes have been reported in literature, affirming the reliability of ileal conduit diversion in patients with neurogenic bladder who are not candidates for continent solutions.^13^ Long-term follow-up will focus on renal function monitoring, stoma care, and surveillance for stomal or anastomotic complications.

We reported an adult male with sacral agenesis and situs inversus who presented with neurogenic bladder and recurrent urinary tract infections. We performed a left-sided ileal conduit due to failed conservative management. Considering the rarity and complexity of such coexisting anomalies, early diagnosis and individualized surgical planning are essential to prevent longterm renal complications and improve quality of life.
